# The Sugarcane Defense Protein SUGARWIN2 Causes Cell Death in *Colletotrichum falcatum* but Not in Non-Pathogenic Fungi

**DOI:** 10.1371/journal.pone.0091159

**Published:** 2014-03-07

**Authors:** Flávia P. Franco, Adelita C. Santiago, Flávio Henrique-Silva, Patrícia Alves de Castro, Gustavo H. Goldman, Daniel S. Moura, Marcio C. Silva-Filho

**Affiliations:** 1 Departamento de Genética, Escola Superior de Agricultura Luiz de Queiroz, Universidade de São Paulo, Piracicaba, SP, Brazil; 2 Departamento de Genética e Evolução, Universidade Federal de São Carlos, São Carlos, SP, Brazil; 3 Faculdade de Ciências Farmacêuticas, Universidade de São Paulo, Ribeirão Preto, SP, Brazil; 4 Laboratório Nacional de Ciência e Tecnologia do Bioetanol (CTBE), Campinas, SP, Brazil; 5 Departamento de Ciências Biológicas, Escola Superior de Agricultura Luiz de Queiroz, Universidade de São Paulo, Piracicaba, SP, Brazil; University of Nebraska-Lincoln, United States of America

## Abstract

Plants respond to pathogens and insect attacks by inducing and accumulating a large set of defense-related proteins. Two homologues of a barley wound-inducible protein (BARWIN) have been characterized in sugarcane, SUGARWIN1 and SUGARWIN2 (*sugarcane wound-inducible proteins*). Induction of *SUGARWINs* occurs in response to *Diatraea saccharalis* damage but not to pathogen infection. In addition, the protein itself does not show any effect on insect development; instead, it has antimicrobial activities toward *Fusarium verticillioides*, an opportunistic fungus that usually occurs after *D. saccharalis* borer attacks on sugarcane. In this study, we sought to evaluate the specificity of SUGARWIN2 to better understand its mechanism of action against phytopathogens and the associations between fungi and insects that affect plants. We used *Colletotrichum falcatum*, a fungus that causes red rot disease in sugarcane fields infested by *D. saccharalis*, and *Ceratocystis paradoxa*, which causes pineapple disease in sugarcane. We also tested whether SUGARWIN2 is able to cause cell death in *Aspergillus nidulans*, a fungus that does not infect sugarcane, and in the model yeast *Saccharomyces cerevisiae*, which is used for bioethanol production. Recombinant SUGARWIN2 altered *C. falcatum* morphology by increasing vacuolization, points of fractures and a leak of intracellular material, leading to germling apoptosis. In *C. paradoxa*, SUGARWIN2 showed increased vacuolization in hyphae but did not kill the fungi. Neither the non-pathogenic fungus *A. nidulans* nor the yeast *S. cerevisiae* was affected by recombinant SUGARWIN2, suggesting that the protein is specific to sugarcane opportunistic fungal pathogens.

## Introduction

Plants respond to pathogens and insect attacks by modulating the expression of a large set of genes, many of which are believed to have a direct role in plant defense [1]. During an herbivore attack, plant defense genes are usually up-regulated, and some of their products inhibit digestive proteases and reduce the nutritional quality of ingested proteins, discouraging additional feeding [2]. In addition, phytopathogens modulate specific signaling pathways, resulting in increased expression of genes coding for PR-proteins (pathogenesis-related proteins), many of which have antimicrobial effects [2]–[11].

BARWIN is a wound- and pathogen-inducible protein that can be isolated from barley seeds and leaves [12], [13]. Two homologues of BARWIN have been identified in sugarcane: SUGARWIN1 and SUGARWIN2 (sugarcane wound-inducible protein) [14], [15]. The *SUGARWINs* are induced in response to methyl jasmonate treatment, mechanical wounding and *Diatraea saccharalis* (Fabricius) attack but are not induced in response to infection by *Fusarium verticillioides* (Sacc.) Nirenberg, an opportunistic fungus. Despite its high expression level in response to *D. saccharalis* attack, the protein has no effect on insect development or mortality [15]. However, SUGARWIN2 has antimicrobial effects on *F. verticillioides*, causing changes in hyphae morphology and leading to cell death by apoptosis [15].

Usually, a *D. saccharalis* borer attack in sugarcane is followed by pathogens that take advantage of the openings left by the borer to colonize the stem. *F. verticillioides,* which causes fusarium rot, and *Colletotrichum falcatum* (Went), which causes red-rot, are highly disseminated in sugarcane crops with *D. saccharalis* infestations, which form borer rot complex in sugarcane [16], [17]. This infestation causes extensive damage to crops, leading to reductions in productivity, and contaminates the sugarcane juice [17]. The soilborne fungus *Ceratocystis paradoxa* (Dade) causes pineapple disease in sugarcane, which is responsible for many losses in sugarcane production [18] and was also used in this work.

Studies have showed associations between insects and fungi that affect plants. In sugarcane, *Fusarium spp.* positively affects the larval survival and development of *Eldana saccharina* [19]. Another example involves the European corn-borer *Ostrinia nubilalis* (Hübner), which grows 20% faster in maize tissues infected with *Colletotrichum graminicola* [20]. A positive interaction between *Leptoglossus occidentalis* and *Diplodia pinea* was identified in *Pinus pinea* [21]. However, symbiotic microbes associated with plants can positively influence plant resistance to herbivores and affect plant vigor [22]–[24]. The association between insects and fungi is important to better understand the role of *SUGARWINS* in the sugarcane defense response once they are induced by the insect and affecting the fungus. It is also important to know the protein specificity and the mechanism of protein action because much sugarcane is used for ethanol production via fermentation. Therefore, it is important to also evaluate the antifungal activity of SUGARWIN2 in yeast, the fungus responsible for fermentation.

The goal of this study was to analyze the specificity of SUGARWIN2 on other sugarcane phytopathogenic fungi (*C. falcatum* and *C. paradoxa*) and two model fungi, *Aspergillus nidulans*, which is non-pathogenic to sugarcane, and *Saccharomyces cerevisiae*, which is eventually responsible for sugarcane juice fermentation. This study is important to better understand the SUGARWIN function in fungi and the relationships between fungi and insects that affect plants.

## Results

### _His_SUGARWIN2 Alters *Colletotrichum falcatum* and *Ceratocystis paradoxa* Mycelial Morphology

**Figure 1:**
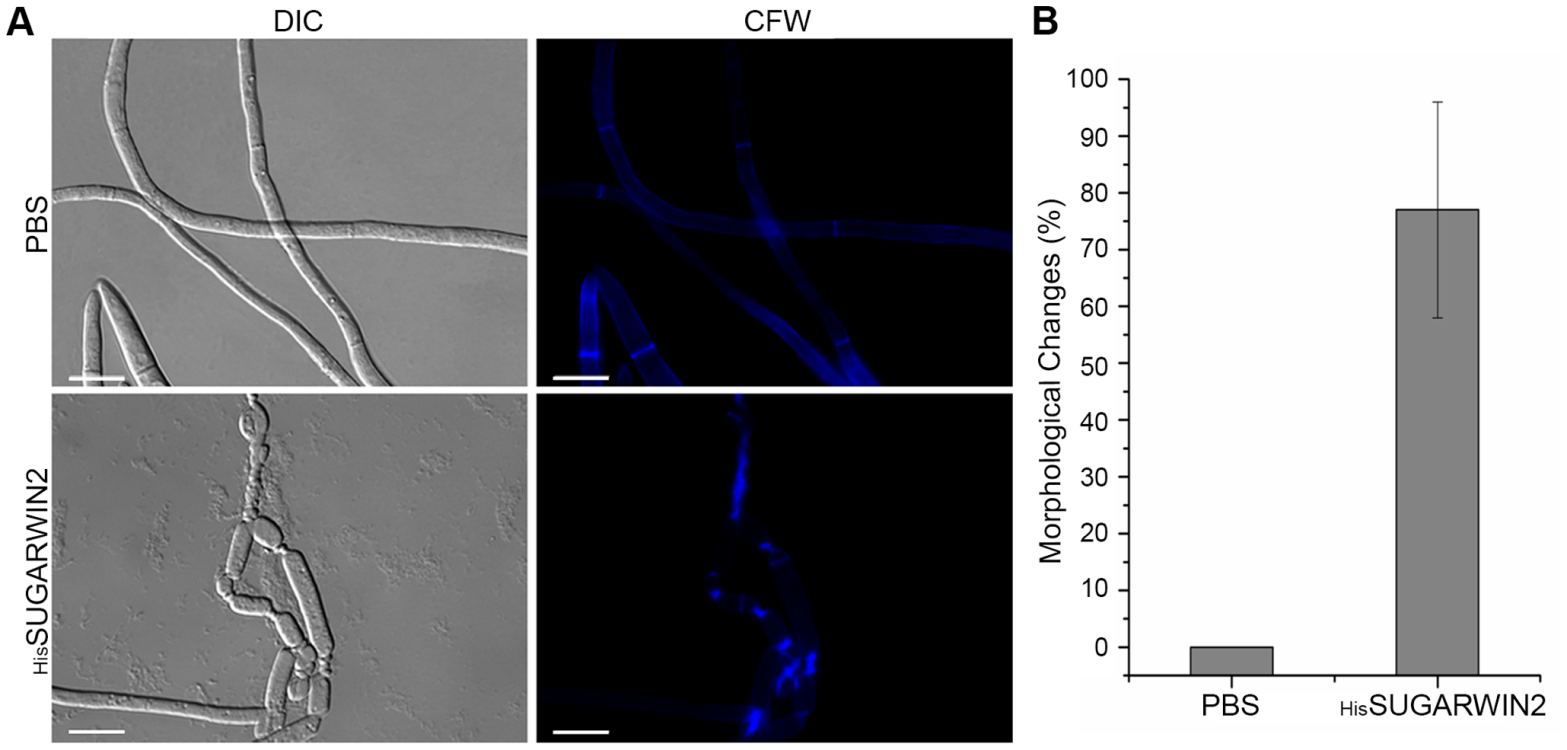
Effects of recombinant sugarcane wound-inducible protein 2 (_His_SUGARWIN2) on the hyphal morphology of *Colletotrichum falcatum*. **A,** Calcofluor assay on *C. falcatum. C. falcatum* germlings were grown in the absence of _His_SUGARWIN2 for 16 h of exposure to phosphate-buffered saline (PBS) at 25°C (control) or in the presence of 160 µM _His_SUGARWIN2 for 16 h at 25°C. CFW = Calcofluor White. The bars represent 10 µm. **B,** Percentage of *C. falcatum* hyphae that showed morphological changes after 160 µM _His_SUGARWIN2 treatment. The bars represent the standard error of the mean percent.

**Figure 2:**
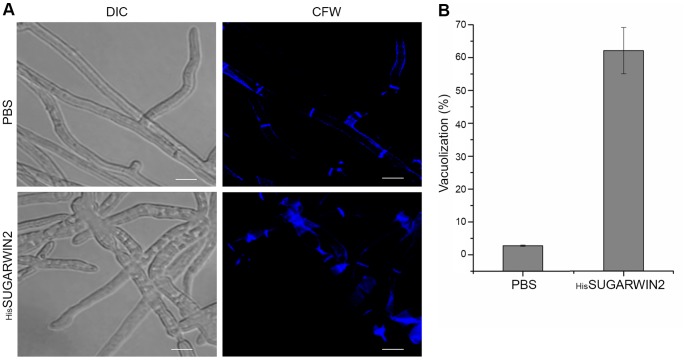
Effects of recombinant sugarcane wound-inducible protein 2 (_His_SUGARWIN2) on the hyphal morphology of *Ceratocystis paradoxa*. **A,** Calcofluor assay on *C. paradoxa. C. paradoxa* germlings were grown in the absence of _His_SUGARWIN2 for 16 h of exposure to phosphate-buffered saline (PBS) at 25°C (control) or in the presence of 160 µM _His_SUGARWIN2 for 16 h at 25°C. CFW = Calcofluor White. The bars represent 10 µm. **B,** Percentage of *C. paradoxa* hyphae that showed morphological changes after 160 µM _His_SUGARWIN2 treatment. The bars represent the standard error of the mean percent.

To test the effect of _His_SUGARWIN2 on *C. falcatum* and *C. paradoxa*, differential interference contrast (DIC) and fluorescence microscopy with Calcofluor White (CFW) analyses were performed. CFW staining was used to verify the integrity of the fungal cell wall due to its chitin binding capacity. Optical brighteners of the diaminostilbene type, such as Calcofluor, seem to be non-toxic and useful in morphological and developmental studies of fungi [25]. The SUGARWIN2 protein, but not SUGARWIN1, was chosen for our experiments because its gene expression in sugarcane plants is nearly 30-fold higher than that of SUGARWIN1 [15]. *C. falcatum* 8-h-old germlings exposed to 160 µM _His_SUGARWIN2 for 16 h showed dramatic morphological changes and an accumulation of chitin in the septa (Fig. 1A). Approximately 77% of these germlings showed abnormalities such as increased vacuolization, multiple points of fractures in the hyphae, and extensive leakage of intracellular material (Fig. 1). Control germlings exposed to phosphate-buffered saline (PBS) only showed normal development and clear fluorescent staining of the septa. Approximately 62% of *C. paradoxa* germlings exposed to 160 µM _His_SUGARWIN2 showed similar morphological changes, especially increased vacuolization (Fig. 2). For quantitative analysis, at least 100 hyphal fragments were counted per sample and were considered as affected if the hyphae had at least one of the symptoms described. Since imidazole and its derivatives were found to have anti-fungal properties, and also found to form hazardous chemicals like hydrogen cyanide, nitrogen oxides etc., we showed that the elution procedure was effective in removing this compound of the purified _His_SUGARWIN2 protein extract (Figure S1).

### Recombinant SUGARWIN2 Causes Cell Death in the Pathogenic Fungus *C. falcatum*

**Figure 3:**
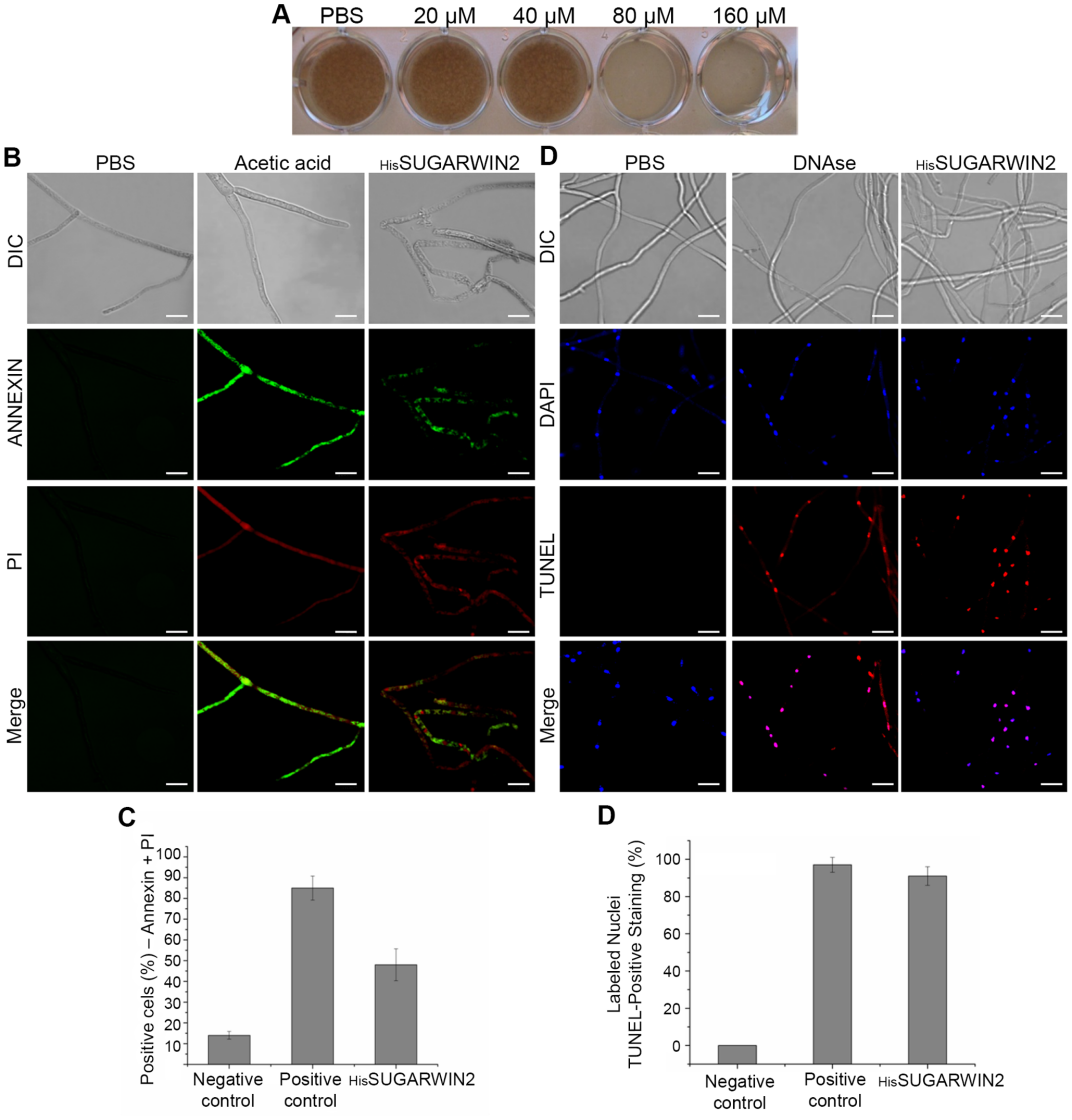
Effects of recombinant sugarcane wound-inducible protein 2 (_His_SUGARWIN2) on cell death in *Colletotrichum falcatum*. **A,** Viability test on *C. falcatum* germlings. *C. falcatum* germlings were treated with different concentrations of _His_SUGARWIN2 (20, 40, 80, and 160 µM) for 16 h. Later, the samples were transferred to new plates containing solid oat medium and incubated at 25°C for another 36 h. **B,** Annexin-V and Propidium Iodide (PI) assay for _His_SUGARWIN2-treated *C. falcatum* germlings. *C. falcatum* germlings grown in the absence of _His_SUGARWIN2 were either exposed to phosphate-buffered saline (PBS), used as a negative control, or grown in the presence of 160 µM _His_SUGARWIN2 for 16 h at 25°C. For the positive control, hyphae were treated with 80 mM acetic acid, pH 3.0, before staining. Germlings were then double-stained with Annexin-V and PI. The bars represent 10 µm. **C,** Percentage of *C. falcatum* hyphae that showed Annexin-V- and PI-positive staining after 160 µM _His_SUGARWIN2 treatment, exposure to PBS (negative control), or acetic acid (positive control). The bars represent the standard error of the mean percent. **D,** Terminal deoxynucleotidyl transferase dUTP nick-mediated end labeling (TUNEL) assay for _His_SUGARWIN2-treated *C. falcatum* germlings. *C. falcatum* germlings grown in the absence of _His_SUGARWIN2 were either exposed to PBS (negative control) or DNAse-treated (positive control). A separate group of germlings was grown in the presence of 160 µM _His_SUGARWIN2 for 16 h at 25°C. The germlings were then fixed and double-stained with 4′,6-diamidino-2-phenylindole (DAPI) and TUNEL. The bars represent 10 µm. **E,** Percentage of *C. falcatum* nuclei that showed TUNEL-positive staining after 160 µM _His_SUGARWIN2 treatment, exposure to phosphate-buffered saline (PBS) (negative control), or DNAse (positive control). The bars represent the standard error of the mean percent.

To test the hypothesis that SUGARWIN proteins are able to affect fungal growth, 8-h-old *C. falcatum* germlings were exposed to increasing concentrations of _His_SUGARWIN2 (20, 40, 80, and 160 µM) for 16 h at 25°C. The viability test shows that _His_SUGARWIN2 treatment at concentrations of 80 and 160 µM causes germling cell death (Fig. 3A). To further investigate the mechanism causing cell death, an Annexin-V and PI assay was used. Annexin-V indicates death by apoptosis due to its ability to bind to phosphatidylserine, which is exposed on cells in early apoptosis [26], while Propidium Iodide (PI) can intercalate with any DNA. However, it cannot penetrate intact cells and cannot mark apoptotic cells unless they are in the final stages of apoptosis when the membrane is already permeable [27]. Thus, cells in early apoptosis are characterized by an increase in the number of Annexin-V-positive [A (+)] cells and PI-negative [PI (-)] cells; late apoptosis (leading to secondary necrosis) is characterized by an increase in the number of [A (+)] and [PI (+)] cells, and primary necrosis is characterized by an increase in the number of [A (-)] and [PI (+)] cells. For a quantitative evaluation of the staining with Annexin-V and PI, at least 100 fragments of hyphae were counted per sample. In this experiment, approximately 14% hyphal fragments [A (+)] and [PI (+)] in the negative control (PBS) and 85% hyphal fragments [A (+)] and [PI (+)] in the positive control were observed. After 16 hours of exposure to 160 µM _His_SUGARWIN2, approximately 48% hyphal fragments [A (+)] and [PI (+)] were observed, suggesting late apoptosis in *C. falcatum* (Fig. 3B and C). Cell death by apoptosis was confirmed by a terminal deoxynucleotidyl transferase dUTP nick-mediated end labeling (TUNEL) assay. This assay uses terminal deoxynucleotidyl transferase to label 3′-OH DNA termini with fluorescein isothiocyanate (FITC)-conjugated dUTP, which can be directly visualized by fluorescence microscopy and then used to identify DNA fragmentation. To evaluate the percentage of TUNEL-positive cells, the nuclei were stained with DAPI (4′,6-diamidino-2-phenylindole). DAPI is a fluorescent stain that binds strongly to AT-rich regions of DNA, resulting in bright blue nuclei. After 16 h of treatment with 160 µM _His_SUGARWIN2, approximately 91% of the nuclei showed TUNEL-positive staining. Untreated control hyphae showed no staining, and the positive control showed 97% TUNEL-positive staining (Fig. 3D and E). The presence of TUNEL-positive cells indicates that cell death in *C. falcatum* occurs by apoptosis [28].

### Recombinant SUGARWIN2 does not Affect the Non-pathogenic Model A*spergillus nidulans*

**Figure 4:**
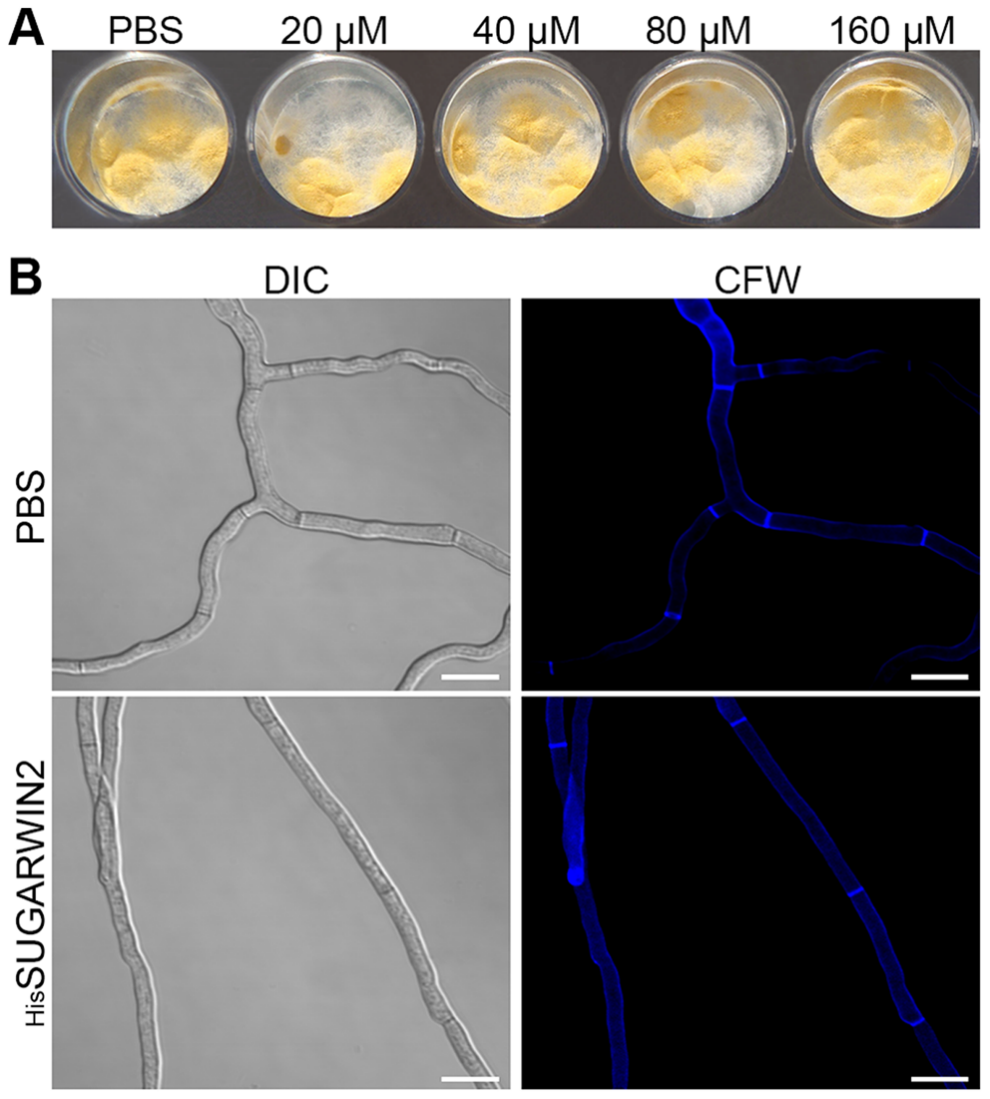
Effects of recombinant sugarcane wound-inducible protein 2 (_His_SUGARWIN2) on *Aspergillus nidulans* germlings. **A,** Viability test on *A. nidulans* germlings. *A. nidulans* germlings were treated with different concentrations of _His_SUGARWIN2 (20, 40, 80, and 160 µM) for 16 h. Later, the samples were transferred to new plates containing solid YAG medium and incubated at 37°C for another 24 h. **B,** Calcofluor assay on *A. nidulans. A. nidulans* germlings were grown in the absence of _His_SUGARWIN2 and exposed to phosphate-buffered saline (PBS) or were grown in the presence of 160 µM _His_SUGARWIN2 for 16 h at 37°C. CFW = Calcofluor White. The bars represent 10 µm.

Eight-hour-old *Aspergillus nidulans* germlings were exposed to increasing concentrations of _His_SUGARWIN2 (20, 40, 80, and 160 µM) for 16 h at 37°C. The viability test showed that _His_SUGARWIN2 treatment does not cause germling cell death at any of the concentrations tested (Fig. 4A). The calcofluor assay also showed no effect of _His_SUGARWIN2 on *A. nidulans*. The germlings exposed to _His_SUGARWIN2 showed normal development, identical to that of germlings exposed to PBS (Fig. 4B). This result indicates that _His_SUGARWIN2 does not affect *A. nidulans* morphology or development.

### Recombinant SUGARWIN2 does not Affect *Saccharomyces cerevisiae*

**Figure 5:**
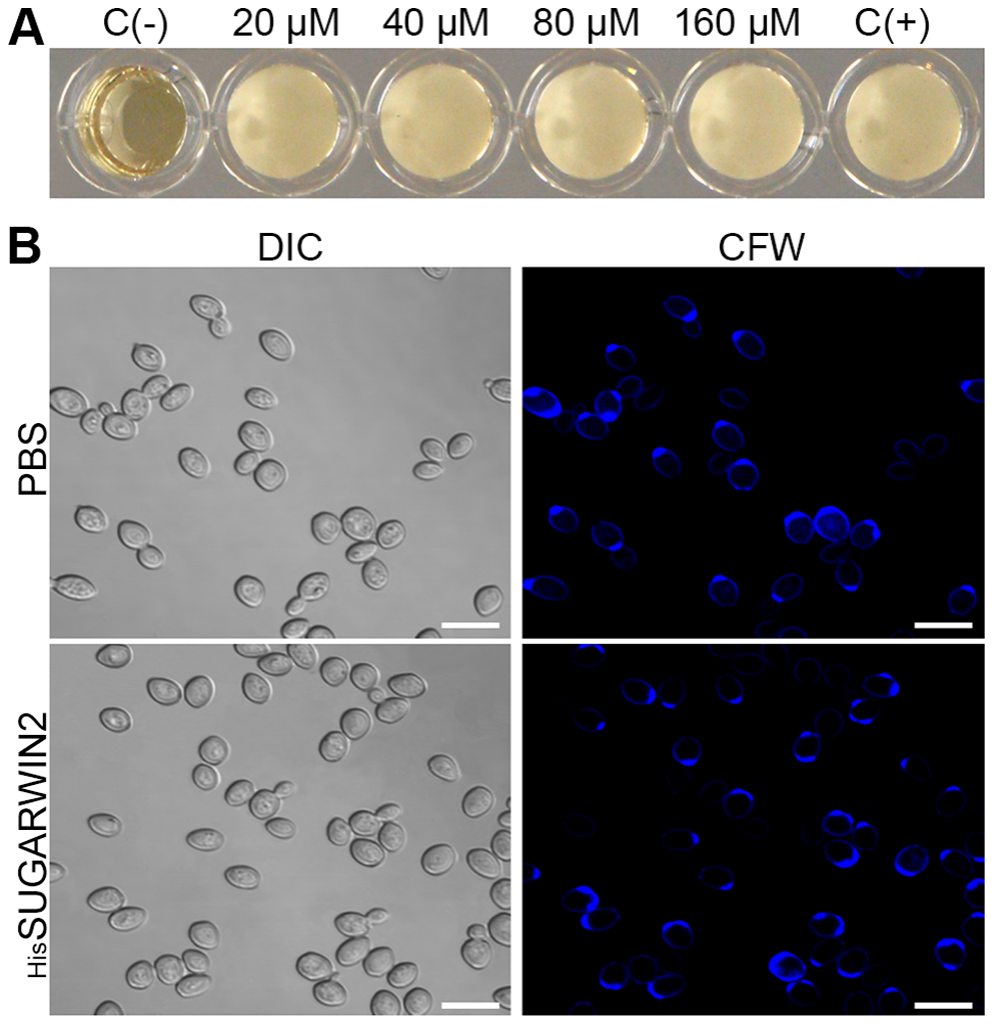
Effects of recombinant sugarcane wound-inducible protein 2 (_His_SUGARWIN2) on *Saccharomyces cerevisiae*. **A,** Viability test on *S. cerevisiae*. *S. cerevisiae* was treated with different concentrations of _His_SUGARWIN2 (20, 40, 80, and 160 µM) for 24 h at 30°C. On the left side, the negative control [C (−)] consisted only of culture medium without yeast and without the protein. On the right side, the positive control [C (+)] consisted of the culture medium with yeast and without protein (PBS was used). **B,** Calcofluor assay on *S. cerevisiae. S. cerevisiae* was grown in the absence of _His_SUGARWIN2 and exposed to phosphate-buffered saline (PBS) or was grown in the presence of 160 µM _His_SUGARWIN2 for 24 h at 30°C. CFW = Calcofluor White. The bars represent 10 µm.

*Saccharomyces cerevisiae* is a model system, and some strains are used for ethanol production owing to their capacity to convert sugar into ethanol [29]. Knowing whether SUGARWIN proteins affect *S. cerevisiae* is important to anticipate problems in the fermentation process in case a transgenic approach is adopted to overexpress the defense protein. *S. cerevisiae* was exposed to increasing concentrations of _His_SUGARWIN2 (20, 40, 80 and 160 µM) for 24 h. The recombinant protein did not show any effect on *S. cerevisiae* (Fig. 5A), indicating that sugarcane juice used for fermentation, even with high levels of SUGARWIN proteins, will neither harm the yeast nor hamper the fermentation process. The calcofluor assay also showed that _His_SUGARWIN2 does not cause any morphological changes in yeast (Fig. 5B).

## Discussion

The plant defense system is under constant selective pressure to synchronously improve its response to pathogens and insects [30]. In this study, we extended the current understanding of the molecular mechanism of SUGARWIN2 action on fungal cell death. We showed that SUGARWIN2 promote *C. falcatum* apoptosis (Fig. 3B and D) in a similar mechanism as that previously described for *Fusarium verticillioides* [15]. We detected changes in *C. falcatum* mycelial morphology when conidia were treated with _His_SUGARWIN2 (Fig. 1). Hyphae abnormalities, the viability of treated cells and TUNEL assay results were also similar to those of *F. verticillioides* [15]. _His_SUGARWIN2 also caused an increase in the vacuolization of *C. paradoxa* (Fig. 2), a soilborne fungus that causes pineapple disease in sugarcane. However, _His_SUGARWIN2 did not kill *C. paradoxa* (data not shown).

*Colletotrichum falcatum* and *Fusarium verticillioides* are consistently associated with stem rot of sugarcane after *D. saccharalis* borer attacks [16], [17], [31]. Recent studies showed that recombinant SUGARWIN2 causes morphological changes and death by apoptosis in *F. verticillioides* [15]. Taken together, these data strongly suggest that SUGARWINs are related to plant defenses against opportunistic pathogens that take advantage of the openings caused by the *D. saccharalis* borer and minimize their damage. Homologues of BARWINs have been shown to exhibit antipathogenic activities against a wide set of plant fungi. *Oryza sativa* OsPR-4b has antifungal activity against *Rhizoctonia solani*. It reduces its growth and distorts and contracts its mycelium [11]. Conversely, the wheat protein WHEATWIN1 inhibits *F. culmorum* growth during spore germination and the elongation of the germ tube in combination with morphological changes such as swelling and shrinkage [5].

The levels of BARWIN proteins in plants may be variable. The levels of WHEATWIN2 and 3 in wheat seeds are 10 µg/g, and the level of WHEATWIN4 is 2 µg/g [7]. It is unclear how the levels of SUGARWINs change in stems of sugarcane attacked by *D. saccharalis*. Further studies should be conducted to elucidate the *in vivo* activity of SUGARWIN. The minimal concentration of SUGARWIN2 protein that showed an effect on fungal growth is greater than the concentration used in previous experiments with other BARWIN proteins [7], [11], [32]. The SUGARWIN proteins may have exhibited a decrease in activity due to the added histidine tag [15].

One intriguing question involves a possible deleterious role of SUGARWINs on *S. cerevisiae* growth because yeast cells are ultimately responsible for the fermentation of sugarcane juice to produce bioethanol [29]. We showed that SUGARWINs have no effect on *S. cerevisiae* growth or viability (Fig. 5A and B). In addition, we showed that _His_SUGARWIN2 had no effect on the hyphae morphology or mortality of *A. nidulans* (Fig. 4A and B), a non-pathogenic filamentous fungus widely used in molecular biology research [33], [34], indicating specificity toward certain sugarcane pathogenic fungi.

The vegetative growth of filamentous fungi occurs through hyphae development, which extends from its apex and branches into the mycelium with septa formation [35]. The septal region displays high chitin synthetase activity [36]. However, yeast (unicellular fungi) grow through a budding mechanism [35]. Thus, the developmental courses of yeast and filamentous fungi are strikingly different, and because SUGARWIN-mediated damage to the septal region is present only in filamentous fungi, developmental differences may explain the lack of effectiveness of _His_SUGARWIN2 on *S. cerevisiae* (Fig. 5).

Several studies have suggested that filamentous growth and conidia formation are controlled by antagonistic mechanisms in fungi [1], [13], [33], [37]–[40]. In particular, two reports showed that the regulatory mechanisms of proteins involved in the cell wall formation of *F. verticillioides* and *A. nidulans* are different [41], [42]. Although the molecular mechanism by which _His_SUGARWIN2 affects the cell wall integrity of *C. paradoxa* (Fig. 2) and *C. falcatum* (Fig. 1), leading to *C. falcatum* cell death by apoptosis (Fig. 3), has not been fully elucidated, the protein is likely involved in a specific mechanism affecting only phytopathogenic fungi and not non-pathogenic fungi such as *A. nidulans,* which showed no morphological changes after recombinant protein treatment (Fig. 4B).

Genes from the BARWIN family are pathogen- and wound-induced and have roles in plant defense [4], [43]–[45]. The *SUGARWIN2* gene is induced by wounding and *D. saccharalis* attack. However, the level of *SUGARWIN2* induced by *D. saccharalis* increases dramatically when compared to the level induced by wounding (approximately 15 times higher expression and approximately 300 times higher expression compared to the control for wounding and *SUGARWIN2* treatment) [15]. This increase in the gene expression level is likely due to constant wounding inflicted on the plant by the borer. Moreover, *SUGARWIN2* expression is local and can be related to the prevention of plant infection by pathogens entering the wound caused by the borer. An increasing number of studies shows associations between insects and fungi [30], [46]–[48]. SUGARWIN2 specificity to pathogenic fungi associated with red rot suggests an unfavorable interaction between *D. saccharalis* and *C. falcatum* because the protein is expressed due to the borer attack and has deleterious effects only on the fungus. Another hypothesis is that the caterpillars somehow benefit from the association with the fungus. In that case, when the plant produces SUGARWIN, it attempts to interfere with this association, reducing fungal infestation and minimizing the damage caused by the possible synergistic interaction between the borer and the fungus.

In this study, we showed SUGARWIN2 specificity toward sugarcane phytopathogenic fungi and its lack of effect on non-pathogenic fungi and yeast. SUGARWIN action has been proposed to be part of the sugarcane strategy against opportunistic fungi that colonize the plant after *D. saccharalis* attack.

## Materials and Methods

### Heterologous Expression of SUGARWIN2

The cDNA coding for the SUGARWIN2 protein was fused to a six histidine tail using the vector pPICZα A from the Pichia expression kit EasySelectTM - Invitrogen [15]. The recombinant protein _His_SUGARWIN2 was expressed in *Pichia pastoris.* A single colony of *P. pastoris* containing the *SUGARWIN2* construct was used to inoculate 10 ml of BMGY medium (1% yeast extract, 2% peptone, 100 mM potassium phosphate buffer (pH 7.0), 1.34% YNB, 4 × 10^−5^% biotin, and 1% glycerol), which was incubated at 30°C until an optical density (OD) at 600 nm of approximately 5 was reached. This culture was used to inoculate 500 ml of BMGY and was grown to an OD of 4 to 5. The cells were harvested by centrifugation at 1,500 × *g* for 5 min, resuspended in 100 ml of BMGY with 0.5% methanol instead of glycerol, and incubated at 28°C. To induce gene expression, methanol was added to each sample every 24 h to maintain a final concentration of 0.75%. After 96 h, the cells were harvested by centrifugation at 1,500 × *g* for 5 min, and the supernatant was passed through a 0.45 µm membrane filter (Millipore, Bedford, MA, U.S.A.). The recombinant proteins in the supernatant were purified by affinity chromatography using Ni-NTA-agarose (Qiagen) pre-equilibrated with purification buffer (10 mM Tris-HCl, pH 8.0; 50 mM NaH_2_PO_4_; and 100 mM NaCl). After binding, the proteins were eluted with two-column volumes of purification buffer containing increasing imidazole concentrations (10, 25, 50, 75, 100, and 250 mM). The fractions containing the _His_SUGARWIN2 protein were dialyzed in phosphate-buffered saline (PBS, pH 7.4) (137 mM NaCl, 2.7 mM KCl, 10 mM Na_2_PO_4_, and 2 mM KH_2_PO_4_) and sterilized with a 0.22 µm filter (Millipore). The protein concentrations were determined using a BCA protein assay kit (Pierce).

### Fungi Treatment with _His_SUGARWIN2 Protein and an Evaluation of its Effects on Cells and Mycelial Morphology

Conidia of *C. falcatum, C. paradoxa* or *A. nidulans* (1.5×10^4^) were inoculated into wells with coverslips of a 24-well plate containing liquid potato dextrose (PD) medium for *C. falcatum* and *C. paradoxa* and liquid yeast glucose (YG) medium [0.5% yeast extract, 2% glucose, and 0.1% trace elements (75 mM ZnSO_4_·7H_2_O, 180 mM H_3_BO_3_, 25 mM MnCl_2_·4H_2_O, 18 mM FeSO_4_·7H_2_O, 6 mM CoCl_2_·5H_2_O, 6 mM CuSO_4_.5H_2_O, 1 mM (NH_4_)_6_Mo_7_O_24_ · 4H_2_O, and 140 mM EDTA) at pH 6.7] for *A. nidulans*. After 8 h of incubation at 25°C (*C. falcatum* and *C. paradoxa*) or 37°C (*A. nidulans*), _His_SUGARWIN2 was added to each well to a final concentration of 160 µM. The plates were then kept at the same temperature for 16 h. The treatments were performed in triplicate. The morphological analysis was performed after 16 h, and PBS was used as a negative control. For *Saccharomyces cerevisiae* treatments, 1.5×10^4^ cells were inoculated into a microtube containing 500 µl of liquid YPD (1% yeast extract, 2% peptone, and 2% glucose) medium and _His_SUGARWIN2 at a final concentration of 160 µM. The microtubes were incubated at 30°C for 24 h, and PBS was used as a negative control. The treatments were performed in triplicate.

For the Calcofluor assay, slides containing *C. falcatum, C. paradoxa*, *A. nidulas* or *S. cerevisiae*, after treatment with _His_SUGARWIN2, were prepared with the addition of 2 µl of a Fluorescent Brightener 28 (Calcofluor White M2R) (Sigma-Aldrich) solution (1 µg/ml) and maintained for 10 min in the dark [5]. The images were acquired using a confocal laser scanning Olympus FV1000 microscope. A DAPI filter was used (excitation at 365 nm and emission at 445/50 nm). The images were analyzed using Olympus Fluoview FV10-ASW software. The treatments were performed in triplicate.

### Viability Test

Conidia of *C. falcatum* and *A. nidulans* were treated with _His_SUGARWIN2 as described above. _His_SUGARWIN2 was added to each well to a final concentration of 20, 40, 80, or 160 µM. PBS was used as a negative control. After 16 h of treatment, all cells were transferred to a 24-well plate containing solid oat medium (*C. falcatum*), BDA medium (*C. paradoxa*) or yeast agar glucose (YAG) medium (*A. nidulans*). The plates were then incubated for an additional 36 h at 25°C for *C. falcatum* and *C. paradoxa* and for an additional 24 h at 37°C for *A. nidulans*. The treatments were performed in triplicate.

For the *Saccharomyces cerevisiae* treatments, 5×10^3^ cells were inoculated into wells of a 96-well plate containing liquid YPD medium with different concentrations of _His_SUGARWIN2 (20, 40, 80 and 160 µM). The negative control consisted only of culture medium without yeast or the protein, and the positive control consisted of the culture medium with yeast and without the protein (PBS was used). Plates were incubated at 30°C for 24 h. The treatments were performed in triplicate.

### Annexin-V and PI Assay

Phosphatidylserine exposure was detected by an annexin-V-Fluos staining kit (Roche) as described by [49] with some modifications. The hyphae were harvested and washed with sorbitol buffer (1.2 M sorbitol, 0.5 mM MgCl_2_, and 35 mMK_2_HPO_4_, pH 6.8). The cell walls were digested with 15 U of lyticase (Sigma) in sorbitol buffer for approximately 15 min at 37°C. The cells were then washed with binding buffer (10 mM HEPES/NaOH, pH 7.4, 140 mM NaCl, and 2.5 mM CaCl_2_) containing 1.2 M Sorbitol (binding-sorbitol buffer). To 96 µl hyphae suspensions in binding-sorbitol buffer, 2 µl of annexin V (Roche) and 2 µl of a propidium iodide (PI) working solution (50 µg/ml) were added, and the mixture was incubated for 15 min at room temperature. The slides were mounted with the hyphae suspensions. For the apoptosis positive control, the cells were treated with 80 mM acetic acid, pH 3.0, for 15 min [50], and for the necrosis positive control, the cells were fixed with a fixative solution (3.7% formaldehyde, 50 mM sodium phosphate buffer, pH 7.0, and 0.2% Triton X-100) for 15 min at room temperature. The images were acquired using a confocal laser scanning Olympus FV1000 microscope. We used a filter for Annexin-V (excitation at 450–500 nm and emission at 515–565 nm) and PI (excitation at 550/25 nm and emission at 605/70 nm). The images were analyzed using Olympus Fluoview FV10-ASW software. The treatments were performed in triplicate.

### Terminal Deoxynucleotidyl Transferase dUTP Nick-mediated End Labeling (TUNEL) Assay

DNA strand breaks were demonstrated by a TUNEL assay using the In Situ Cell Death Detection Kit, TMR red (Roche Vol. 25, No. 5, 2012/623 Diagnostics GmbH, Mannheim, Germany). After 16 h of treatment with _His_SUGARWIN2 at 25°C, as previously described, the supernatants containing the hyphae were transferred to microtubes and washed with PBS. The TUNEL assay was then performed as described previously, with minor modifications [28]. The hyphae were first fixed with a fixative solution for 30 min at room temperature and then washed in PBS. The hyphae were incubated in a digestion solution (lyticase at 1 mg/ml) for 1 h at 37°C, followed by washing with PBS. The hyphae were then incubated in permeabilization solution (0.1% Triton X-100 and 0.1% sodium citrate) for 10 min on ice, followed by a wash with PBS. The hyphae were next incubated with a TUNEL reaction solution for 1 h at 37°C, followed by a wash with PBS. The cells were then subjected to nuclear staining for 3 min with DAPI (Sigma-Aldrich) at 0.1 µg/ml. The positive control was treated with 10 U of DNAse (Fermentas) for 1 h at 37°C before TUNEL treatment. The images were acquired using a confocal laser scanning Olympus FV1000 microscope. We used a filter for DAPI (excitation at 365 nm and emission at 445/50 nm) and TUNEL (excitation at 550/25 nm and emission at 605/70 nm). The images were analyzed using Olympus Fluoview FV10-ASW software. The treatments were performed in triplicate.

## Supporting Information

Figure S1 Imidazole treatment does not interfere in hyphae morphology. *C. falcatum* or *C. paradoxa* germlings was exposed to phosphate-buffered saline (PBS) (control) or the fungus grown in the presence of imidazole 100mM dialyzed in PBS for 16 h at 25°C. The bars represent 10 µm.
(TIF)
