# Supplementary figures and images for: The Sugarcane Defense Protein SUGARWIN2 Causes Cell Death in Colletotrichum falcatum but Not in Non-Pathogenic Fungi
Source: PLoS One. 2014 Mar 7;9(3):e91159. doi: 10.1371/journal.pone.0091159 (PMC3946703; doi:10.1371/journal.pone.0091159)

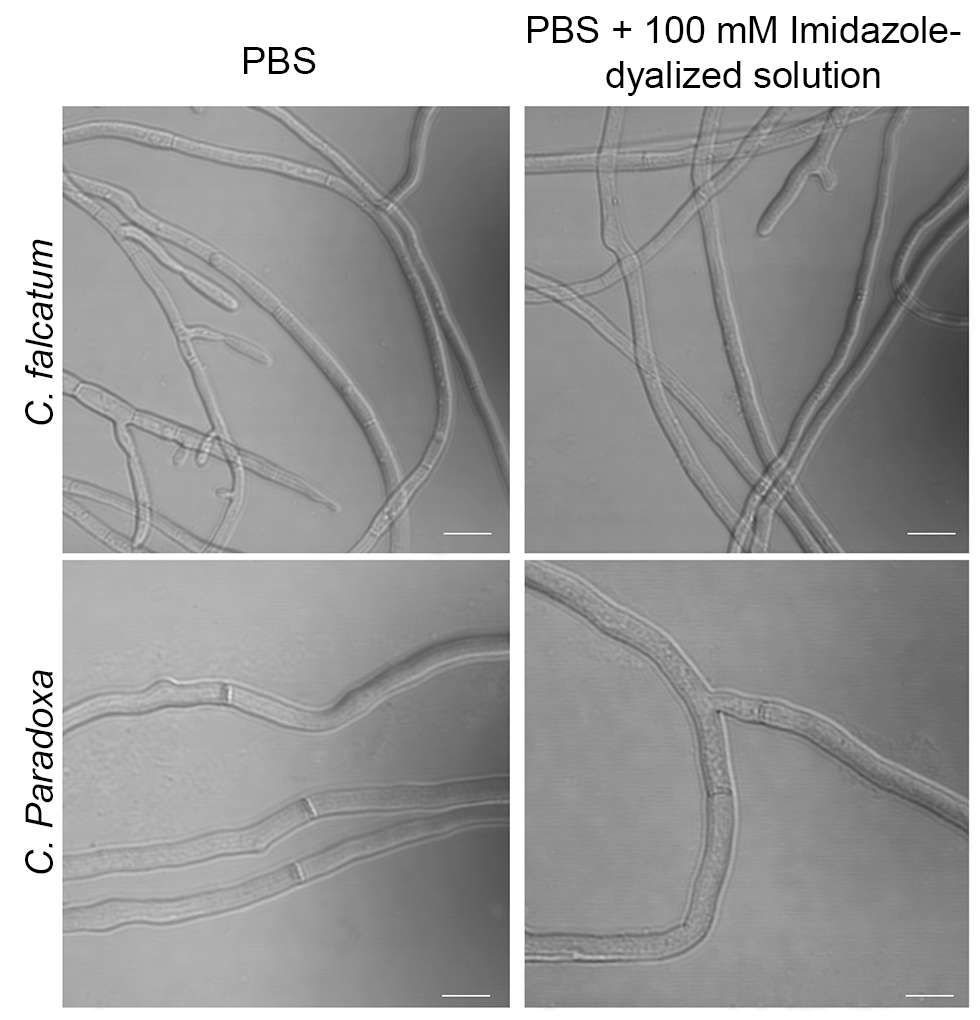

Supplement: Figure S1 — Imidazole treatment does not interfere in hyphae morphology. C. falcatum or C. paradoxa germlings was exposed to phosphate-buffered saline (PBS) (control) or the fungus grown in the presence of imidazole 100mM dialyzed in PBS for 16 h at 25°C. The bars represent 10 µm. (TIF) [file pone.0091159.s001.tif]
